# Understanding the geographical burden of stunting in India: A regression‐decomposition analysis of district‐level data from 2015–16

**DOI:** 10.1111/mcn.12620

**Published:** 2018-05-23

**Authors:** Purnima Menon, Derek Headey, Rasmi Avula, Phuong Hong Nguyen

**Affiliations:** ^1^ Poverty, Health and Nutrition Division International Food Policy Research Institute (IFPRI) Washington DC USA

**Keywords:** child undernutrition, decomposition analysis, determinants, India, spatial analysis, stunting

## Abstract

India accounts for approximately one third of the world's total population of stunted preschoolers. Addressing global undernutrition, therefore, requires an understanding of the determinants of stunting across India's diverse states and districts. We created a district‐level aggregate data set from the recently released 2015–2016 National and Family Health Survey, which covered 601,509 households in 640 districts. We used mapping and descriptive analyses to understand spatial differences in distribution of stunting. We then used population‐weighted regressions to identify stunting determinants and regression‐based decompositions to explain differences between high‐ and low‐stunting districts across India. Stunting prevalence is high (38.4%) and varies considerably across districts (range: 12.4% to 65.1%), with 239 of the 640 districts have stunting levels above 40% and 202 have prevalence of 30–40%. High‐stunting districts are heavily clustered in the north and centre of the country. Differences in stunting prevalence between low and high burden districts were explained by differences in women's low body mass index (19% of the difference), education (12%), children's adequate diet (9%), assets (7%), open defecation (7%), age at marriage (7%), antenatal care (6%), and household size (5%). The decomposition models explained 71% of the observed difference in stunting prevalence. Our findings emphasize the variability in stunting across India, reinforce the multifactorial determinants of stunting, and highlight that interdistrict differences in stunting are strongly explained by a multitude of economic, health, hygiene, and demographic factors. A nationwide focus for stunting prevention is required, while addressing critical determinants district‐by‐district to reduce inequalities and prevalence of childhood stunting.

**Key messages**

- India carries a high burden of child stunting, but lack of disaggregated stunting data at the district level has been a challenge for policy and program strategies in a decentralized governance system.
- This is the first study to use district‐level data from a recently released national survey to highlight spatial differences in stunting across 640 districts in India.
- Our findings highlight the range of factors that explain differences between high and lower stunting burden districts.
- These results emphasize the importance of focused strategic planning and action to address multiple, and different, district‐specific determinants of stunting across India.

## 1 INTRODUCTION

As a marker of poor nutrition, stunting in early childhood is strongly associated with numerous short‐term and long‐term consequences, including increased childhood morbidity and mortality (Black et al., 2013), delayed growth and motor development (Grantham‐McGregor et al., 2007), and long‐term educational and economic consequences (Dewey & Begum, 2011). In recognition of the high social and economic costs of stunting, the Sustainable Development Goals explicitly include reductions in global stunting, and many countries have adopted the World Health Assembly target of achieving a 40% reduction in stunting by 2025.

Achieving this reduction on a global scale, however, requires rapid progress against stunting in India, which accounts for approximately one third of the world's total population of stunted preschoolers (De Onis, Blössner, & Borghi, 2011). Understanding the underlying determinants of stunting in India—which has long been characterized as having unusually high stunting rates relative to its economic development (Ramalingaswami, Jonson, & Rohde, 1997)—has therefore been the subject of considerable investigation. An array of studies from many disciplines has drawn attention to the multifactorial nature of the problem of stunting in India. Explanations have addressed issues such as economic growth and agricultural production (Fenske, Burns, Hothorn, & Rehfuess, 2013; Headey, Chiu, & Kadiyala, 2012; Subramanyam, Kawachi, Berkman, & Subramanian, 2011), poor sanitation and open defecation (Fenske et al., 2013; Spears, Ghosh, & Cumming, 2013), discrimination against women and girls (Jayachandran & Pande, 2015), poor maternal undernutrition before and during pregnancy (Coffey, 2015), exceptionally poor infant and young child feeding practices (Menon, Bamezai, Subandoro, Ayoya, & Aguayo, 2015), and broader dietary deficiencies (Deaton & Dreze, 2008).

Some previous studies have shown that child undernutrition clusters in specific regions in developing countries (Fenn, Morris, & Frost, 2004; Gebreyesus, Mariam, Woldehanna, & Lindtjorn, 2016) and different types of spatial analysis studies have been conducted to identify geographical inequalities in child stunting (Fenn et al., 2004, Gebreyesus et al., 2016, Adekanmbi, Uthman, & Mudasiru, 2013, Alemu, Ahmed, Yalew, & Birhanu, 2016). However, much less has been done on explaining the factors that contribute to spatial variability in stunting (Di Cesare et al., 2015; Haile, Azage, Mola, & Rainey, 2016; Sharaf & Rashad, 2016; Srinivasan, Zanello, & Shankar, 2013), particularly in India. Although India is a highly populated country with a high burden of stunting, limited evidence exists on spatial analysis to examine the patterns of stunting across the country. To our knowledge, two previous assessments have been done; one at the state level (Cavatorta, Shankar, & Flores‐Martinez, 2015) and another that utilized data from a subset of Indian districts (112 of 640) from a privately conducted survey to examine the role of sanitation (Spears et al., 2013). The paucity of analysis on the geography of stunting in India is problematic for two reasons. First, there are significant economic, social, and cultural differences both across and within states that might well explain the stark geographical disparities in nutrition previously observed in India (Cavatorta et al., 2015). Second, although Indian governance has traditionally been dominated by federal and state governments, the past 20 years has seen a major push to decentralize decision making to the district and subdistrict levels. Hence, a more granular assessment of the differences in stunting across India's 640 districts is essential for targeting and planning purposes.

In this study, we address this knowledge gap with an analysis of a new district‐level data set created to address three research questions: (a) How do stunting prevalence and absolute numbers of stunted children vary across Indian states and districts? (b) Which determinants of stunting are associated with district stunting prevalence? and (c) Which determinants account for the variation in stunting observed across high‐ and low‐stunting districts?

## 2 METHODS

### 2.1 Data

This paper utilizes a district‐level data set generated from National Family Health Survey (NFHS)‐4 Fact Sheets (International Institute for Population Sciences, 2017) and the 2011 Census of India (Ministry of Home Affairs, 2012). The NFHS‐4 survey is unique in being the first national survey to provide data on stunting that is representative at the district level for all 640 districts spread across 36 states. NFHS‐4 was conducted from January 20, 2015 to December 4, 2016, gathering data from 601,509 households. The survey covered topics such as child anthropometrics, parental education levels, household demographics, and access to health and sanitation services. The fact sheets from all 640 districts were released on April 2017, but unit level data have not been released (as of November 2017). These district fact sheets provide summary data on 114 indicators including stunting and its key determinants. We supplemented these indicators with data from the Census of India (Ministry of Home Affairs, 2012), including estimates of the population aged 0–5 years, open defecation density, ownership of household durables, and housing characteristics.

### 2.2 Measures

Our outcome indicator of interest is the district level stunting prevalence, which is the proportion of children 0–59 months of age who have their height‐for‐age two standard deviations below the World Health Organization (WHO, 2006) growth reference (HAZ < −2). The key determinants of stunting in India were selected based on conceptual frameworks from the previous literature, particularly UNICEF (1990) and the Lancet Nutrition Series (Bhutta et al., 2013). The UNICEF framework distinguishes between **immediate** determinants (diets and disease burdens) and **underlying** determinants. The Lancet framework links these determinants to interventions, noting that **nutrition‐specific** interventions address immediate determinants, whereas interventions and policies in **nutrition‐sensitive** sectors address underlying determinants. In this paper, we distinguish between i**mmediate determinants**, **nutrition‐specific interventions**, and **underlying** determinants.

The **immediate determinants** included indicators related to maternal undernutrition and child feeding practices. We used women's low body mass index (BMI < 18.5 kg/m^2^) as a proxy for maternal undernutrition. Indicators for infant and young child feeding included early initiation of breastfeeding (proportion of infants 0–23 months who were breastfed within 1 hr of birth), exclusive breastfeeding (the proportion of infants 0–5.9 months of age who fed only breast milk), timely introduction of complementary foods (proportion of children 6–8.9 months of age who were introduced solid and semi‐solid foods), and adequate diet (proportion of children 6–23 months old who received four or more food groups and a minimum meal frequency). Some of these variables are only available for subsets of districts.

The **nutrition‐specific interventions** included antenatal care (ANC) during the first trimester, adequate ANC (at least four ANC visits), and iron and folic acid (IFA) consumption (at least 100 IFA during the last pregnancy). Indicators related to infant's postnatal care included full immunization, vitamin A supplementation, and oral rehydration solution during diarrhoea. Although some of these are health care interventions, they are considered nutrition‐specific interventions because they act as important platforms for delivery of nutrition‐specific interventions such as micronutrient supplements and nutrition counselling and reach households in the first 1,000 days of life.

The **underlying determinants** examined included mother's education (≥10 years of schooling), age at marriage (at 18 years or older), sanitation, an asset index, and household size. For sanitation, we used water within premises (with the assumption that more access to water may facilitate more hygienic practices) and open defecation density (the number of people estimated to engage in open defecation per square kilometre). An asset index was constructed from district‐level data, using the first principal component extracted from 19 different variables, including housing structure, house ownership, presence of a kitchen, access to electricity, clean cooking fuel, assets, and access to a bank account. We also included the proportion of scheduled caste/tribes (designated groups of historically disadvantaged people in India) in the district because it is an important dimension of inequality in India.

### 2.3 Statistical analyses

Several complementary methods of analysis were applied to these data. We first estimate the absolute numbers of stunted children by multiplying the stunting prevalence with the estimated number of children 0–5 years of age from the Census of India. We mapped stunting prevalence by district to graphically analyse patterns of stunting across India. We tabulated stunting prevalence and absolute numbers of stunted children by states and by three major state groupings (northern states, southern states, and north‐eastern and island states). District stunting prevalence was then categorized into four bins based on current WHO cut‐off values for public health significance (WHO, 2010): low prevalence (<20%), moderate prevalence (20–29.9%), high prevalence (30–39.9%), and very high prevalence (≥40%). The differences in determinants were tested for statistical significance across these different stunting burden categories, using analysis of variance and Bonferroni post hoc comparisons.

Second, to identify the determinants of stunting prevalence at the district level, we examined the bivariate associations between stunting and various determinants using scatter plots and tested for normality of the distributions using the Kolmogorow–Smirnov test. Three variables (4+ antenatal visits, open defecation density, and asset scores) were not normally distributed and showed non‐linear bivariate relationships with stunting; hence, they were log‐transformed. Multivariate linear regression was then used to examine the different factors associated with stunting. For this regression analysis, we dropped a few variables that were either highly correlated with another variable (e.g., ANC in the first trimester was highly correlated with 4+ ANC visits) or were only available for a subset of the districts (exclusive breastfeeding, timely introduction of foods, and oral rehydration solution during diarrhoea were only available for 425, 186, and 328 districts, respectively). Because we are primarily interested in explaining differences across districts rather than differences across states, all models included state‐fixed effects, meaning that we are analysing within‐state variation in stunting prevalence. We therefore report both total *R* ^2^, but also the within‐ and between‐state coefficients of determination. All regression models were weighted by the population of children under 5 years because the district population sizes vary substantially. In terms of specifications, we first estimated bivariate models for each variable. We then estimated a multivariable model including only immediate determinants and nutrition‐specific interventions and then estimated a full multivariable model that included underlying determinants. In addition to gauging whether the coefficients on immediate determinants are robust to potential confounding factors, this approach allows us to investigate potential causal pathways by examining how coefficients on immediate determinants change as underlying determinants are added to the model (MacKinnon, Krull, & Lockwood, 2000).

In the last step of our analysis, we applied a regression‐decomposition to assess the ability of the various determinants described above to predict spatial patterns in stunting and differences between very high‐burden and low‐burden districts. This approach has been used widely in literature to study mean outcome differences between groups (Jann, 2008), including differences in child malnutrition between geographical areas (Sharaf & Rashad, 2016; Spears et al., 2013; Srinivasan et al., 2013) and between populations measured at different points of time (Headey, Hoddinott, Ali, Tesfaye, & Dereje, 2015). This analysis effectively combines the analysis of differences in means of the explanatory variables (*X*) and regression estimates of the coefficients associated with these variables (β_*X*_). Specifically, the “explained” difference between one spatial unit (District A) and another unit (District B) is the product of the difference in the mean of *X* across the two samples (*X*_A_ − *X*_B_) and the coefficient of *X* from a pooled regression model (β_*X*_). Intuitively, if a particular *X* variable has a large regression coefficient (“marginal effect”) and a large difference in means over two districts, then this variable will play a large role in explaining the interdistrict difference in stunting. An attractive feature of the decomposition approach is that it gauges the ability of all the variables in the model to predict interdistrict differences, as well as the ability of the model as a whole to account for these differences. In this analysis, we implemented a decomposition at means of the stunting differences between very high‐burden (stunting > 40%) and low‐burden districts (stunting < 20%) with the objective of understanding how high‐burden districts can move towards much lower rates of stunting. We report the share of actual stunting accounted for by this decomposition, as well as the share unexplained by the model as a whole.

## 3 RESULTS

**Figure 1:**
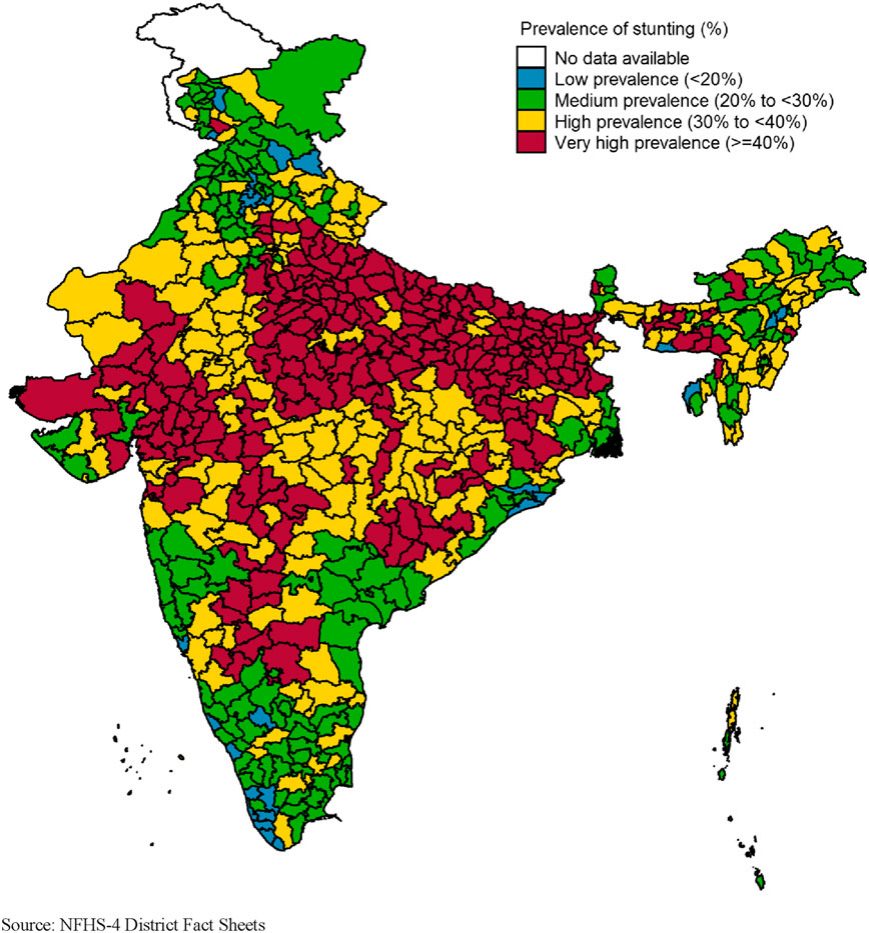
Maps of stunting prevalence in Indian districts, 2015–2016

India achieved a sizeable improvement in stunting between 2006 and 2016, with a decline from 48.0% to 38.4% among children below 5 years (International Institute for Population Sciences, 2017). Despite this, stunting in India remains high and variable across districts, ranging between 12.4% and 65.1% (Figure 1). In total, there are more than 63 million children stunted in the country, which is more than one third of the global estimate for 2013 (De Onis & Branca, 2016). Stunting varies substantially across major regions and states, both in terms of prevalence and absolute numbers of stunted children (Table 1). The populous northern states of India contain approximately 52.6 million stunted children, accounting for more than 80% of stunted children in the country. Average district stunting prevalence for these states varies from 25.2% in Himachal Pradesh to 48.2% in Bihar and 46.3% in Uttar Pradesh. These latter two states are very large, containing 9.2 million and 14.3 million stunted children, respectively. In comparison, all of the Southern states collectively contain 8.1 million stunted children and the north‐eastern and island states some 2.4 million. Even so, stunting prevalence in these other regions is relatively high in many instances, with one third of children in Andhra Pradesh and Karnataka estimated to be stunted, for example. Among reasonably populous states, only Kerala had an average district stunting prevalence below the 20% threshold.

**Table 1:** Stunting prevalence and population stunted, by major regions and states of India

	# districts	District stunting prevalence (%)			Population stunted
		Mean	Range (min, max)		
Northern states	**442**	**35.5**			**52,623,659**
Bihar	38	48.2	35.6	57.3	9,208,676
Chandigarh	1	28.7	28.7	28.7	34,278
Chhattisgarh	18	38.9	30.6	49.0	1,368,203
Gujarat	26	39.4	22.6	50.6	2,991,236
Haryana	21	32.4	19.8	52.3	1,141,734
Himachal Pradesh	12	25.2	18.4	30.3	203,373
Jammu & Kashmir	22	27.4	18.2	43.1	541,625
Jharkhand	24	45.0	38.5	59.4	2,434,078
Madhya Pradesh	50	42.0	32.1	52.1	4,549,506
Maharashtra	35	35.2	21.3	47.6	4,561,180
NCT of Delhi	9	31.6	22.5	38.6	656,792
Odisha	30	34.8	15.3	47.5	1,811,802
Punjab	20	25.3	17.6	34.8	786,316
Rajasthan	33	39.1	28.4	54.3	4,146,682
Uttar Pradesh	71	46.3	32.2	65.1	14,300,000
Uttarakhand	13	31.4	22.9	39.1	449,780
West Bengal	19	32.7	23.3	45.5	3,438,399
Southern states	**105**	**26.9**			**8,128,073**
Andhra Pradesh	13	31.2	22.1	44.1	1,624,603
Goa	2	19.9	18.3	21.4	28,873
Karnataka	30	35.3	18.6	55.8	2,596,295
Kerala	14	19.2	12.4	27.7	689,068
Puducherry	4	26.6	19.0	32.0	31,701
Tamil Nadu	32	27.0	17.2	37.0	2,022,964
Telangana	10	29.4	15.7	38.3	1,134,569
North‐east and islands	**93**	**31.0**			**2,404,214**
Andaman & Nicobar	3	24.3	20.1	32.5	9,692
Arunachal Pradesh	16	29.4	20.5	42.0	63,165
Assam	27	35.3	24.6	47.4	1,686,136
Dadra and Nagar	1	41.7	41.7	41.7	21,223
Daman and Diu	2	28.1	18.9	37.3	6,282
Lakshadweep	1	27.0	27.0	27.0	1,959
Manipur	9	31.0	21.0	37.1	111,542
Meghalaya	7	40.1	16.8	51.6	242,762
Mizoram	8	29.6	23.7	36.9	47,720
Nagaland	11	28.4	18.7	41.8	81,906
Sikkim	4	30.8	24.0	42.3	19,651
Tripura	4	26.5	19.5	32.5	112,176
Total	**640**	**36.0**			**63,155,946**

*Note*. NCT = National Capital Territory.

The bold font means the overall number for the regions (eg. Northern States, Southern States, ect).

Across all 640 districts in India, 239 districts have stunting prevalence in excess of 40% (very high), and 441 districts have stunting prevalence between 30% and 40% (high; Table 2). Only 29 districts have stunting levels between 10% and 20%, and most of these are in South India. Although there is considerable clustering of stunting within states, intrastate variance in district stunting prevalence is still reasonably high. Specifically, inter‐state variation explains 56% of the variation in district stunting prevalence (see Table 4 below); hence, 44% of variation in interdistrict stunting prevalence is accounted for by intrastate variation.

**Table 2:** Stunting prevalence and absolute numbers of stunted children, by stunting burden categories

	No. districts	Share of districts (%)	Stunting rate (%)	Stunted children	Share of stunted children (%)
Stunting burden categories					
Low prevalence (<20%)	29	4.5	16.9	723,651	1.1
Medium prevalence (20–29.9%)	170	25.6	25.9	8,872,991	14.1
High prevalence (30–39.9%)	202	31.6	35.2	16,363,830	25.9
Very high prevalence (≥ 40%)	239	37.3	46.9	37,179,537	58.9
Total	640	100.0	38.8	63,140,011	100.0

National averages and district variability for various determinants across stunting burden categories of stunting are presented in Table 3. On average, nearly a quarter of women have low BMI. More than 40% of children were breastfed within an hour of birth, and only 55% were exclusively breastfed. Moreover, complementary feeding is of great concern with less than 10% of children receiving an adequately diverse diet. In case of underlying determinants, more than a third of women had at least 10 years of education, and two thirds of girls married after the age of 18. Open defecation is still prevalent in more than half of the population. Coverage is above 50% for several nutrition‐specific interventions. More than half of the women received ANC in the first trimester or had at least four ANC visits, but only 30% of the women consumed at least 100 IFA during pregnancy. Coverage of full immunization and vitamin A supplementation was nearly 60%. There was high interdistrict variability for most determinants across stunting burden category districts (Table 3). The most inequity among districts is observed for women's low BMI, women's education (≥10 years), asset score, ANC, and IFA consumption where the high‐burden stunting districts have levels that are 2–3 times lower than the low‐burden districts, and gaps range from 16% to 40%.

**Table 3:** Differences in stunting prevalence and its determinants across stunting burden categories

	Overall prevalence	Low prevalence (<20%)	Medium prevalence (20–<30%)	High prevalence (30–<40%)	Very high prevalence (≥40%)
Stunting		16.9	25.9	35.3	46.9
Immediate determinants					
Women with BMI <18.5	22.9	12.5^a^	15.3^a^	21.9^b^	28.6^c^
Initiated breastfeeding early	41.6	52.5^a^	49.7^a^	47.1^a^	39.3^b^
Exclusive breastfeeding^a^	54.9	48.7^a^	59.0^a^	59.7^a^^,^ b	53.3^a^^,^ c
Timely introduction of foods^b^	42.7	29.5^a^	63.2^b^	46.9^c^	34.2^a^
Adequate diet	9.6	17.3^a^	15.2^a^	9.8^b^	6.9^c^
Nutrition‐specific interventions					
ANC first trimester	58.6	77.2^a^	67.9^b^	62.3^c^	50.7^d^
4+ ANC visits	51.2	74.4^a^	67.5^a^	54.5^b^	35.9^c^
Taken IFA during pregnancy	30.3	46.8^a^	42.4^a^	33.1^b^	20.2^c^
Full immunization	62.0	75.7^a^	67.7^a^	62.6^b^	56.7^c^
Received vitamin A in last 6 months	60.2	72.4^a^	66.0^a^	58.7^b^	55.5^b^
ORS during diarrhoea^c^	50.6	91.4^a^	65.7^a^	57.0^b^	48.5^c^
Underlying determinants					
Women with ≥10 years school	35.7	56.1^a^	44.0^b^	33.9^c^	25.7^d^
Married after age of 18	73.2	90.3^a^	83.0^b^	76.5^c^	68.5^d^
Asset score (scale 0–100)	36.0	57.0^a^	55.4^a^	50.9^b^	46.9^c^
Water within premises	42.3	60.7^a^	50.4^a^	40.1^a^^,^ b	36.3^a^^,^ b
Open defecation density (km^2^)	252.8	130.4^a^	142.7^a^	187.7^a^	400.9^b^
Scheduled caste population	14.9	13.4	14.7	14.4	15.5
Household size	5.0	4.5^a^	4.6^a^	4.9^a^^,^ b	5.4^b^

*Note*. Significant differences (*p* < .05) between groups are denoted by different subscript letters. ANC = antenatal care; BMI = body mass index; IFA = iron and folic acid; ORS = oral rehydration solution.

a Data for exclusive breastfeeding are available for 425 districts only.

b Data for timely introduction of foods are available for 186 districts only.

c Data for ORS during diarrhoea are available for 328 districts only.

Bivariate analysis indicates that stunting is associated with a wide range of immediate and underlying determinants (Table 4). The strongest associations were observed for asset scores (β = −10.6 and −16.6 for Quintile 4 and 5, respectively) and low BMI in women (β = −0.73, 95% CI [0.66, 0.79]). The districts with higher coverage of nutrition specific‐interventions had lower prevalence of stunting (β ranged from −0.27 to −0.17).

**Table 4:** Multivariate linear regression models of stunting among children 0–5 years of age against its underlying determinants, with state‐fixed effects

	Bivariate model		Partial model^a^		Full model^b^	
	Coefficient	[95% CI]	Coefficient	[95% CI]	Coefficient	[95% CI]
Women with BMI <18.5	0.86****	[0.79, 0.94]	0.54****	[0.46, 0.62]	0.30****	[0.21, 0.40]
Initiated breastfeeding early	−0.24****	[−0.29, −0.20]	0.05	[−0.00, 0.10]	0.02	[−0.03, 0.07]
Adequate diet	−0.55****	[−0.64, −0.46]	−0.21****	[−0.31, −0.11]	−0.22****	[−0.32, −0.13]
4* ANC visits, log	−1.30****	[−1.41, −1.20]	−0.36****	[−0.53, −0.18]	−0.17*	[−0.36, 0.02]
IFA during pregnancy	−0.35****	[−0.38, −0.32]	−0.06**	[−0.11, −0.00]	0.02	[−0.03, 0.08]
Full immunization	−0.26****	[−0.30, −0.22]	−0.05**	[−0.09, −0.00]	−0.00	[−0.04, 0.04]
Received vitamin A in last 6 months	−0.24****	[−0.28, −0.19]	−0.02	[−0.07, 0.02]	−0.03	[−0.07, 0.01]
Women with ≥10 years school	−0.44****	[−0.49, −0.40]			−0.14****	[−0.22, −0.07]
Married after age of 18	−0.33****	[−0.38, −0.28]			−0.09***	[−0.14, −0.04]
Water within premises	−0.11****	[−0.14, −0.07]			−0.02	[−0.05, 0.02]
Asset score, Quintile 1	0.00	[0.00, 0.00]			0.00	[0.00, 0.00]
Asset score, Quintile 2	−2.32**	[−4.27, −0.37]			−1.91**	[−3.45, −0.37]
Asset score, Quintile 3	−5.17****	[−7.05, −3.29]			−2.92***	[−4.84, −1.00]
Asset score, Quintile 4	−10.57****	[−12.56, −8.59]			−2.99**	[−5.44, −0.54]
Asset score, Quintile 5	−16.58****	[−18.54, −14.61]			−3.43**	[−6.47, −0.39]
Log open defecation density	0.52****	[0.45, 0.58]			0.11***	[0.03, 0.18]
Scheduled caste population	0.05	[−0.05, 0.15]			−0.04	[−0.12, 0.03]
Household size	7.01****	[6.18, 7.84]			1.90****	[0.88, 2.92]
*R* ^2^, total			.70		.74	
*R* ^2^, between‐state			.56		.56	
*R* ^2^, within‐state			.32		.41	
*N*			635^c^		635	

*Note*. All models included state‐fixed effects and are weighted by the number of children 0–5 years in each district. ANC = antenatal care; BMI = body mass index; IFA = iron and folic acid.

a Partial model included immediate and nutrition‐specific interventions.

b Full model included all factors such as immediate and underlying determinants as well as nutrition‐specific interventions.

c Data for final model were available for 635 districts; 5 districts were excluded due to lack of data on full immunization.

* *p* < .10.

** *p* < .05.

*** *p* < .01.

**** *p* < .001.

In the partial multivariable regression analyses (Table 4), which only includes immediate determinants and nutrition‐specific interventions, we found significant relationships between women's BMI and adequate diet among children with stunting. For every 1‐percentage point increase in women with low BMI, there is an associated 0.54 percentage point increase in stunting. Districts with higher proportion of children with adequate diet had lower stunting prevalence (β = −0.21, 95% CI [−0.31, −0.11]). In terms of nutrition‐specific interventions, higher coverage of ANC (4 + ANC visits) had a large and statistically significant negative association with stunting (β = −0.36, 95% CI [−0.53, −0.18]), and IFA consumption had a much smaller association (β = −0.06, 95% CI [−0.11, −0.01]).

In the full model, where all the determinants were included together, all of the above associations (except for IFA consumption) remained significant; however, the magnitude of the coefficients decreased, suggesting that various underlying determinants explain variation in factors such as ANC and maternal BMI. For example, the coefficient on maternal BMI declines from 0.53 in the partial model to 0.30 in the full model, and the coefficient on ANC declines from −0.36 to −0.22. Interestingly, the coefficient on adequate diet is essentially unchanged. For both models, the total *R* ^2^ coefficients are high (0.70 and 0.74), although this is partly because state‐fixed effects explain 56% of the national variation in district stunting prevalence. In terms of the explanatory power of the various determinants, the more relevant statistic is the within‐state *R* ^2^ which shows that the explanatory variables in the partial and full models explain 31% and 42%, respectively, of the within‐state variation in district stunting prevalence.

**Figure 2:**
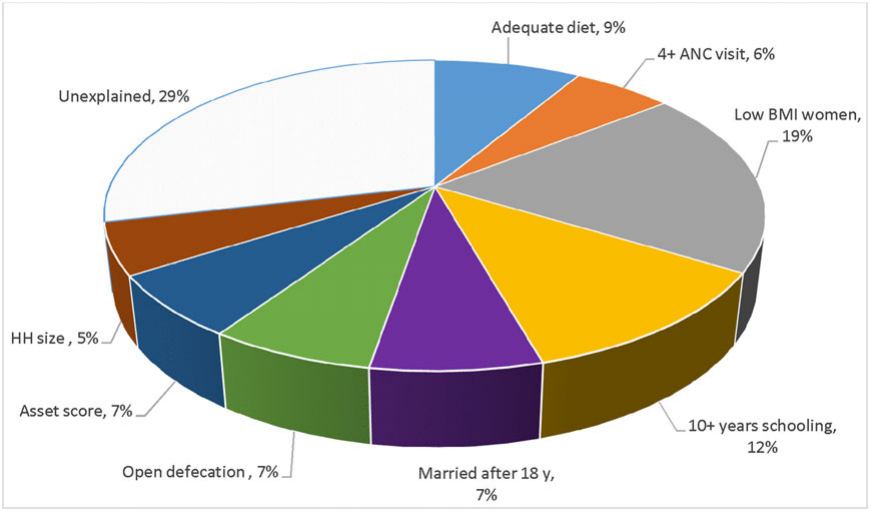
Factors contributing to the difference in stunting prevalence between very high‐burden (stunting > 40%) and low‐burden districts (stunting < 20%). ANC = antenatal care; BMI = body mass index; HH = household

The variables selected in the full regression model were used in the decomposition analysis to estimate the extent to which differences in these factors explained differences in stunting prevalence across very high‐ and low‐burden districts. Overall, the decomposition models performed well, explaining 71% of the observed differences in stunting prevalence between high‐ and low‐burden districts (Figure 2). This explained share is accounted for by the differences in women's low BMI (19%), women's education (12%), adequate diet among children (9%), asset scores (7%), open defecation (7%), age at marriage (7%), ANC (6%), and household size (5%). Decomposition analyses comparing low‐ and medium‐burden districts found similar results (results not shown).

## 4 DISCUSSION

In parallel with global attention and political commitment to reducing undernutrition, India has made considerable progress in reducing child malnutrition in the last decade. However, stunting prevalence remains high and extremely variable across districts and particularly high in populous northern states. High‐stunting districts are characterized by lower levels of immediate and underlying determinants and low levels of nutrition‐specific intervention coverage. The key factors associated with stunting were women's BMI, women's education, women's age at marriage, coverage of ANC, adequacy of child diets, household assets, and open defecation. These results suggest that if very high‐stunting districts could catalyse improvements in these social, economic, and dietary factors, they would eliminate 71% of the gap with low stunting districts.

Our analysis has several unique strengths. Previous studies have applied decomposition techniques to understand stunting differences between poor‐performing states and a single high‐performing state (Tamil Nadu) using child level data from NHFS‐III (Cavatorta et al., 2015) and to understand changes in India's national stunting prevalence between NHFS‐I (1992/1993) and NHFS‐III (2005–2006; Headey, Hoddinott, & Park, 2016). Our study uses the most recent data, is comprehensive in examining spatial variation across the entire country, and geographically granular in that it focuses on interdistrict variation in stunting in a country with tremendous spatial variation in nutrition and its proximate and underlying determinants. Geographical clustering of stunting in India is pronounced, as is the clustering of various immediate and underlying determinants and intervention coverage. These determinants account for around three quarters of the differences in stunting prevalence between the very high and low prevalence districts. A geographical lens, therefore, highlights spatial dimensions of undernutrition that might be overlooked in child‐level analyses. These findings also offer insights on the kinds of gaps that must be closed with equity‐enhancing, geographically targeted policy instruments and high‐quality implementation of these instruments. Our analysis, therefore, provides timely evidence for policymakers to tackle stunting in India, in a context of India's commitments to the global nutrition targets and the Sustainable Development Goals.

We acknowledge some of the limitations of this analysis. The cross‐sectional and geographically aggregated nature of our data means that our analysis is ecological in nature and could still be hampered by confounding factors. The richer unit level data from the NHFS‐4, which at the time of writing had still not been released, will permit a more extensive analysis. We were unable to examine changes in linear growth outcomes by different age categories, which can provide important clues about the aetiology of stunting. From a policy perspective, however, there is significant merit in understanding district‐level variation, because the district is an increasingly important unit in India's ongoing decentralization process and a district‐level focus is central to India's newly launched National Nutrition Strategy (NITI Aayog, 2017). Moreover, despite the more ecological nature of our analysis, our findings are well‐aligned with those from many other studies that have examined the determinants of stunting in India, using both unit‐level and district‐level data sets. For instance, almost all previous analyses of stunting determinants find strong associations with mother's education (Alderman & Headey, 2016). Several studies also link stunting in India to monotonous diets (Menon et al., 2015) and poor sanitation (Spears et al., 2013), even after controlling for wealth and parental education. Other studies have also found ANC visits in their last birth to be strongly associated with stunting in South Asia (Headey et al., 2016). A final limitation of note is that we were not able to examine relationships between all aspects of infant and young child feeding practices and child stunting because they are age‐specific indicators (exclusive breastfeeding (EBF) for children 0–5 months and timely introduction of foods for children 6–8 months), and data were only available for a subset districts.

A focus on addressing women's nutrition emerges as a key priority area in our analyses, similar to other studies of malnutrition in South Asia (Coffey, 2015). We find, for instance, that low women's BMI explained almost a fifth of the difference between high‐ and low‐burden stunting districts, corroborating results from previous studies that maternal undernutrition before and during pregnancy is a major determinant of poor fetal growth and child stunting (Black et al., 2013). Accounting for one fifth of the global population with 42% of low BMI prepregnant women (Coffey, 2015), India faces a critical challenge because preconception undernutrition among women can influence birth outcomes and child growth through influencing early placental and embryonic development, epigenetic effects, and competition for nutrients between mother and baby (King, 2016).

Including maternal BMI, variables reflecting women's well‐being—BMI, education, early marriage, and access to ANC—explain close to half the difference between high and low stunting districts. Discrimination against women is a widely suspected cause of India's unusually high rate of stunting, including small size at birth and low birth weight (Coffey, 2015). Although the variables in our analysis do not capture gender discrimination in terms of man–woman or boy–girl differences, the indicators used reflect several investments in girls and women—education levels, age at marriage, maternal nutrition, and use/access to ANC services. These indicators of investments in girls and women are likely to have both biological and social pathways to better nutrition for children. For example, early marriage, and consequently early child bearing, is more likely to lead to preterm births or small for gestational age births and perhaps also higher fertility prevalence over the life course (Branca, Piwoz, Schultink, & Sullivan, 2015; Temmerman, Khosla, Bhutta, & Bustreo, 2015).

Our study has significant policy implications. The high burden of stunting across most districts in India implies that strategies to address stunting must be rolled out across most of India, and a narrow spatial targeting is unlikely to deliver radical reductions in stunting. Moreover, the fact that 44% of interdistrict variation in stunting prevalence is explained by intrastate variation suggests that decentralization of the district level is critical. In addition to the intrastate variation, inter‐state differences were also prominent (56% of the variation in district stunting was explained by state‐fixed effects). This is likely due to vast differences across states in administrative and governance approaches, implementation capabilities, and economic and sociocultural differences.

The regression model used in this study has significant predictive power, suggesting that the variables used in this analysis could be used for monitoring multisectoral initiatives to reduce stunting. These initiatives should prioritize improving the socioeconomic, nutritional, and health status of girls and women—their nutrition, education, early marriage, and access to care during and after pregnancy—and improvements in sanitation and overall socioeconomic status of the household. We note, however, that many of these factors are rooted in social and cultural contexts that will require more holistic societal changes than policy instruments alone can deliver.

In conclusion, our findings reiterate the complex and multifaceted nature of the burden of stunting in India. The granular district‐focused analysis in this study, a first for India, highlights the concentration of this burden in the northern and eastern regions and the close associations between stunting and a wide range of nutrition‐specific and nutrition‐sensitive factors. The most important policy implications of our analysis are the need for a stunting prevention focus that is nationwide but focused on addressing critical determinants district‐by‐district to reduce inequalities and the prevalence of childhood stunting.

## CONFLICTS OF INTEREST

The authors declare that they have no conflicts of interest.

## CONTRIBUTIONS

PM conceived the manuscript, reviewed the analyses, and wrote significant sections of the manuscript. DH conducted the statistical analysis and wrote major sections of the manuscript. RA conducted the literature review, reviewed, and revised the manuscript. PHN conducted the statistical analysis and wrote significant sections of the manuscript. All authors read and approved the final submitted manuscript.
